# Fabrication of collagen-based biomaterials for sports medicine application

**DOI:** 10.1093/rb/rbaf116

**Published:** 2025-11-13

**Authors:** Bingqian Jiang, Zhi Zhou, Chengxuan Yu, Huizhu Li, Shunyao Li, Chenquan Hua, Runhe Huang, Zhengnan Xia, Bin Kong, Jun Chen

**Affiliations:** Department of Sports Medicine, Huashan Hospital, Fudan University, Shanghai 200040, China; Department of Sports Medicine, Huashan Hospital, Fudan University, Shanghai 200040, China; Department of Sports Medicine, Huashan Hospital, Fudan University, Shanghai 200040, China; Department of Sports Medicine, Huashan Hospital, Fudan University, Shanghai 200040, China; Department of Sports Medicine, Huashan Hospital, Fudan University, Shanghai 200040, China; Department of Sports Medicine, Huashan Hospital, Fudan University, Shanghai 200040, China; Department of Sports Medicine, Huashan Hospital, Fudan University, Shanghai 200040, China; Department of Orthopedics, Fengyang County People’s Hospital, Anhui 233100, China; Faculty of Physical Education, Fudan University, Shanghai 200434, China; Department of Sports Medicine, Huashan Hospital, Fudan University, Shanghai 200040, China

**Keywords:** collagen biomaterials, sports injury regeneration, extracellular matrix engineering, medical device

## Abstract

As the principal constituent of the extracellular matrix, collagen exhibits significant therapeutic potential in sports medicine, owing to its distinct triple-helical configuration and inherent biocompatibility. This biomaterial serves as a foundational material for scaffolds, membranes, patches and dressings targeting tendon repair, cartilage reconstruction and bone defect remediation. However, its clinical translation was hampered by limitations: poor tensile strength risks mechanical failure under load, immunogenicity from residual epitopes can trigger adverse reactions and rapid enzymatic degradation compromises structural integrity before tissue maturation. This review elucidates current properties and resources of collagen-based biomaterial and critically analyzes its inherent limitations and their clinical consequences. It emphasizes how evolving tissue engineering strategies directly mitigate barriers. Molecular crosslinking and chemical modification are employed to enhance tensile properties and delay degradation, critical for mechanically demanding environments. Composite blending with polymers compensates for mechanical weakness while retaining bioactivity. Advanced processing techniques such as 3D printing and electrospinning enable precise fiber alignment, replicating native tissue anisotropy and improving functional outcomes. Rigorous decellularization protocols further mitigate immunogenicity. This review further examines recent preclinical and clinical progress in collagen-based biomaterials for tendon, ligament, cartilage and bone regeneration, highlighting successful translations and ongoing challenges. Future directions focus on refining these strategies to accelerate the development of next-generation, clinically robust collagen therapies for sports medicine.

## Graphical abstract

**Figure:**
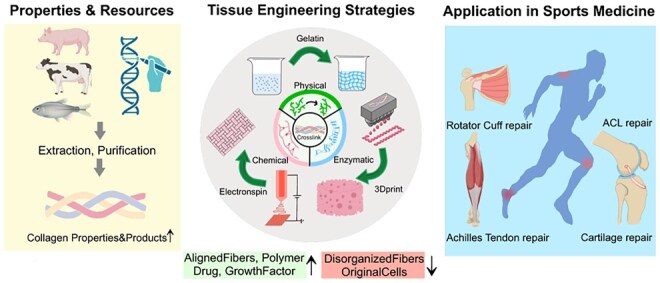

## Introduction

Sports medicine, which focuses on musculoskeletal injury management through prevention, treatment and functional restoration, confronts the central clinical challenges in reconstructing load-bearing tissues under dynamic mechanical loading conditions. The extracellular matrix (ECM) of these load-bearing tissues, with collagen as its primary structural component, determines the tensile strength and functional integrity of the tissue through its hierarchical fiber arrangement and mechanical properties [1]. Traumatic injuries induce structural discontinuities in collagen fibril continuity and homeostatic dysregulation, whereas conventional interventions result in autograft morbidity, allogeneic immunogenicity and mechanical mismatch [2, 3]. Collagen-based biomaterials offer multifaceted advantages that hold promise for overcoming difficulties associated with the treatment of sports injuries.

**Figure 1.**
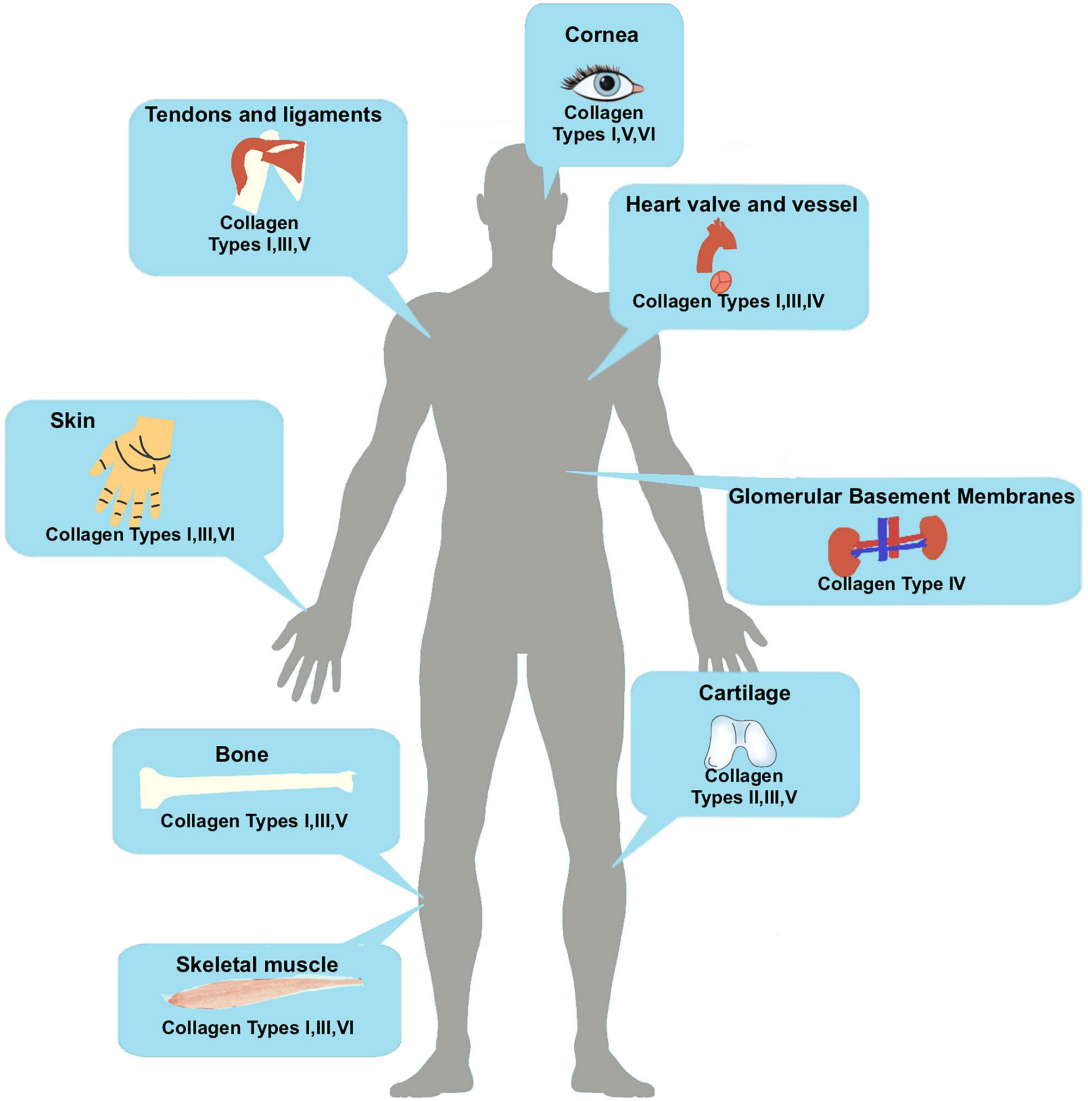
Schematic of distribution of collagen in human body. Col I accounts for approximately 80% of the total collagen and is involved in the formation of tissues such as skin, tendons, ligaments, bones and cartilage in the human body.

The tissue distribution characteristics of collagen are closely related to its dynamic musculoskeletal applications. As a fibrous protein with a unique triple-helical tertiary structure, collagen accounts for 25–30% of the total proteome mass in mammalian systems. This protein constitutes 60–70% of the ECM components, forming a fundamental framework that maintains the structural integrity of the ECM through its hierarchical organizational structure [4–6]. Fibrous collagen (types I, II, III, V and XI) accounts for over 90% of the total collagen in the human body, with type I collagen (Col I) predominating in load-bearing tissues (80–85%) [7]. Col I, encoded by the *COL1A1/COL1A2* genes, comprises 80% of total collagen and is the main component of the ECM in skin tissue (70–80% dry weight), bone tissue (90% of the organic matrix) and fibrocartilage tissue. The distribution of collagen in the human body is shown in Figure 1.

Furthermore, this protein exhibits notable biomaterial properties including mechanical stability, tissue compatibility, osteoinductive capacity and promotion of wound healing. Owing to its high homology with natural ECM, excellent biocompatibility and controllable regenerative induction ability, collagen has become an irreplaceable biomaterial in sports medicine [8]. The value of the collagen products depends on their biomimetic characteristics. Col I scaffolds can precisely simulate the fibrously oriented structure of tendons and provide a matrix environment conducive to cartilage and bone defect repair [9–11]. Type II collagen (Col II) composite hydrogels maintain chondrocyte phenotypes and inhibit fibrotic repair [12, 13]. Acellular collagen matrices optimize the biological microenvironment of the graft–bone interface by preserving ECM active components [14, 15]. In addition, sports medicine demands a high dynamic load tolerance from the repair materials. Through crosslinking modification, nanocomposite technology and 3D printing with directed alignment, the tensile strength and degradation stability of collagen-based materials have been significantly improved, meeting the repair needs of high-pressure environments such as rotator cuff. These breakthroughs not only address the mechanical performance limitations of traditional collagen products but also advance their applications in clinical scenarios, such as arthroscopic minimally invasive surgery and personalized tissue engineering. Recently, the National Medical Products Administration approved an increasing number of collagen-based products for use in sports medicine (Table 1).

**Table 1.** List of collagen-based product related to sports medicine approved by National Medical Products Administration (https://www.nmpa.gov.cn/, date of research: 23 April 2025)

Tissues	Proprietary name	Main collagen type	Form	Manufacturers	Ref
Cartilage	COLTRIX CartiRegen	Porcine type I collagen	Injectable scaffold	Ubiosis Co., Ltd	[16]
Bone	Smartbone ORTHO	Bovine type I collagen	Filling material	Industrie Biomediche Insubri S.A.	[17]
	Collagen Dental Bone Filler	Porcine type I collagen	Filling material	GENOSS Co., Ltd.	[18]
	Geistlich Mucograft	Type I collagen	Implantable membrane	Geistlich Pharma AG	[19]
Tendon	CuffPair: Tendon repair matrix	Type I Bovine Achilles Tendon Collagen	Patch	Beijing Bangsai Technology Co., Ltd.	[20]
	REGENETE Bioinductive Implant System Gen 3.5	Type I Bovine Achilles Tendon Collagen	Patch	Smith&Nephew Inc.	[21]

This review systematically examines the biomechanical properties of collagen to frame recent advancements in medical collagen biomaterials, focusing on four engineering strategies: the crosslinking method, polymer blending during fabrication, collagen fiber alignment and decellularization protocols. It, then, outlines the current status of clinical trials involving the intervention of collagen-based biomaterials. Furthermore, this article reviews its clinical application in the repair and regeneration of tendons, ligaments, cartilage and bone. The last section presents perspectives.

## Collagen properties and resources

In most connective tissues, collagen fibers and their supramolecular assemblies represent a major component of the ECM, forming a hierarchically structured 3D framework that supports cellular organization. Based on its hierarchical structure, collagen exhibits key biological properties, including biocompatibility, hydrophilicity, low immunogenicity, biodegradability, cytocompatibility, supporting cellular adhesion, proliferation, differentiation and migration, along with pro-regenerative capabilities [22]. (i) From a materials science standpoint, collagen-derived biomaterials possess tailorable mechanical properties (including flexibility and tunable elastic modulus), thermal stability and enzymatic resistance. Furthermore, these materials can form hydrogels via physiological triggers such as pH and temperature changes [1, 23–26]. (ii) From a biological perspective, collagen undergoes enzymatic degradation by endogenous proteases, exhibiting biocompatibility, *in vivo* absorption and synergistic interactions with bioactive molecules [27–29]. For example, collagen-based biomaterials exhibit tissue inductivity for tendon and ligament regeneration via mechanotransduction pathways [30]. Collagen scaffolds demonstrate osteoinductive capacity and vascularization potential in bone tissue engineering [31]. Hydrogel scaffolds based on Col I and Col II possess good biological activity, can support the growth of chondrocytes, enhance the expression of chondrocyte-specific genes such as *Sox9*, and repair osteochondral defects through pathways including the inhibition of the TGF-β-Smad1/5/8 signaling pathway [13, 32]. While Col II scaffolds are promising for cartilage regeneration, blending of Col I and Col II aims to balance mechanical strength and chondroinductive properties [33, 34]. Combination of different collagens promotes the integration of surrounding tissues and reduces the risk of immune rejection [13, 35, 36]. In addition, collagen harbors cell-adhesive motifs that regulate cellular morphology, adhesion, migration and differentiation, thus, playing a significant role in cancer signaling [37]. The hierarchical scaffold architecture of collagen mediates hemostasis and induces monocyte/fibroblast chemotaxis via synergistic interactions with fibronectin and growth factors, thereby promoting granulation tissue formation. Collagen also plays critical roles in cutaneous repair by regulating macrophage polarization, epithelial cell proliferation and neovascularization [22]. In general, collagen represents an ideal scaffolding material for applications as both a tissue filler and matrix-dense tissue engineering construct.

Collagen procurement methods are primarily divided into (i) extraction from animal tissues (e.g. porcine bone, bovine hide) and (ii) recombinant production via host cells, including bacteria, yeast and insect cells [38]. Each approach has its own distinct advantages, limitations and processing requirements. Terrestrial mammals, particularly cows and pigs, are the primary sources of collagen in industrial and biomedical fields [39–42]. Although bovine and porcine collagen demonstrate low immunogenicity due to homology with the human ECM, zoonotic disease risks from livestock sources present significant challenges for product development. Recent advancements have highlighted marine organisms, including marine and freshwater fish bones, skin, fins and scales as promising alternative sources of collagen. Marine-derived collagen eliminates zoonotic disease transmission risks while demonstrating reduced immunogenicity and attenuated inflammatory responses [43]. The standard collagen isolation protocol involves three sequential stages: tissue pretreatment, collagen extraction and subsequent purification. Extraction methodology critically determines the structural and functional integrity of collagen macromolecules. The predominant extraction protocol includes neutral salt solutions, acid hydrolysis and enzymatic digestion using proteases, which are achieved through the precise modulation of the solution pH and ionic strength within the tissue matrix [44, 45]. Emerging methodologies, including ultrasonic cavitation, electrodialysis separation and isoelectric focusing, are being developed to preserve the structural integrity and bioactivity [46]. Xu *et al.* combined enzymatic hydrolysis with ultrafiltration to purify type V collagen from bovine cornea. Highly purified collagen, exhibiting a complex triple-helix structure, can inhibit fibroblast proliferation and promote migration [47].

Engineered collagen analogs effectively mitigate immunogenic reactions and eliminate the pathogenic contamination risks inherent to native collagen sources. *KOD,* a collagen-mimetic peptide, is a prototypical biomimetic material that replicates the triple-helical architecture of collagen. Kumar *et al.* engineered synthetic fibrous polymers mimicking the triple-helical conformation of collagen and subsequently applied this nanofibrous collagen-mimetic peptide as a biomimetic thrombus-modulating scaffold [48]. Recombinant humanized type III collagen (RHC III) is a frequently produced collagen variant with extensive commercial applications. Extensive clinical investigations have established a robust biosafety profile and demonstrated its therapeutic potential in cutaneous repair and tissue regeneration. Unlike native type III collagen, RHC III lacks a triple-helical structure, enabling fibroblast endocytosis that enhances endogenous Collagen III biosynthesis while facilitating wound closure and inhibiting hypertrophic scarring [49, 50]. Nevertheless, the key limitations of recombinant production include elevated costs, low yields and lack of essential cofactors and enzymes in the pore-forming system. Recent clinical trials in North America and China have mainly employed RHC III for dermatological aesthetics [51]. Currently, novel approaches are being investigated to facilitate collagen biosynthesis. Hu *et al.* successfully engineered a collagen variant that incorporated adhesive and functional domains using transgenic techniques. The team generated a collagen variant that exhibited superior biocompatibility, thermal stability and structural tunability [52]. Zhou *et al.* used a polyether sulfone (PES) scaffold seeded with human mesenchymal stem cells (hMSCs) to produce matrices composed mainly of Col I and III collagen, proteoglycans and glycoproteins. Following purification and lyophilization, human ECM-like collagen (hCol) was generated, which improved the migration and differentiation of human adipose stem cells(hASCs) in adipogenic induction medium (AIM). *In vitro* experiments confirmed its positive effect on wound healing in mouse [53].

## Tissue engineering strategies for collagen

Collagen demonstrates the previously discussed production advantages, with extraction and purification methodologies reaching advanced maturity [39,40, 44, 45, 54–57]. Notwithstanding these advancements, notable challenges persist in collagen research and development, hampering its practical application. Collagen derived from biological sources for practical applications typically experiences substantial deterioration in mechanical strength, thermal stability and enzymatic resistance, thus, requiring chemical or structural modifications for biomedical translation [58–60]. Current strategies for collagen tissue engineering, including crosslinking, blending strategies, fiber alignment protocols and decellularization methodologies will be discussed in detail below.

### Crosslinking

Industrial applications frequently employ combinatorial crosslinking techniques to achieve collagen functionalization, thereby facilitating the self-assembly and gelation processes. Crosslinking methodologies typically involve chemical, physical and biological modalities that are frequently integrated into multiple stages of collagen-based biomaterial fabrication. Synthetic agent-mediated chemical crosslinking is the most widely adopted strategy for enhancing the properties of biomaterials.

#### Chemical crosslinking

Chemical crosslinking with synthetic agents is the most prevalent strategy aimed at improving the properties of biomaterials. Chemical crosslinking entails covalent modification of the carboxyl and amino functional groups of collagen using carefully selected crosslinking reagents. This methodology facilitates the creation of collagen hydrogels via polymer-chain crosslinking. While enabling rapid hydrogel formation, this approach risks residual crosslinking agents persisting in the hydrogel matrix despite washing steps [61]. Glutaraldehyde (GA), a pioneering crosslinking agent, is widely used in traditional collagen modification protocols because of its notable reactivity and cost-effectiveness. The dialdehyde structure of GA enables Schiff base formation with amine groups from adjacent collagen molecules, effectively bridging the polypeptide chains. Despite its utility, GA-based crosslinking generates residual byproducts and unreacted monomers that resist complete removal, posing risks of localized cytotoxicity and inflammatory responses [61]. Therefore, the process confers enhanced mechanical stability, but residual cytotoxicity from unreacted agents and degradation byproducts represents a critical limitation [62–64]. Alternative reagents, including 1-ethyl-3-(3-dimethylaminopropyl)carbodiimide(EDC)/N-hydroxysuccinimide(NHS), Genipin and Dialdehyde Starch (DAS), offer viable alternatives to GA by combining reduced cytotoxicity with comparable crosslinking efficacy [65–67]. EDC/NHS catalyzes amide bond formation between the carboxyl groups of glutamic/aspartic acid residues in collagen and the amine groups. EDC transforms into water-soluble urea derivatives during crosslinking, thereby preventing residual accumulation within the collagen matrix. The byproducts and unreacted reagents remained water-soluble, facilitating efficient removal via distilled water washing following EDC/NHS crosslinking.

However, chemically synthesized crosslinking agents inevitably pose potential toxicity risks. To address this problem, crosslinking agents extracted from plants have been developed. Genipin, a natural product derived from Gardenia jasminoides fruit, contains reactive functional groups. Its structure includes hydroxyl and ester moieties, which are capable of covalent interactions with amino acid residues in proteins. While preserving the native architecture of collagen, genipin enhances biostability through covalent crosslinking, although this process increases the manufacturing expenses [67]. DAS, generated via the periodate oxidation of natural starch, mediates crosslinking between the amine and imino groups of collagen as a polyaldehyde macromolecule. Recent advances in nano-crosslinking strategies address collagen’s inherent mechanical weaknesses. For instance, dialdehyde cholesterol-modified starch nanoparticles (DACSNPs) form homogeneous Schiff-base linkages with collagen fibers, achieving a 70.8 kPa compressive strength which reaches only 7.8 kPa for pure collagen and reducing gelation time to 15 min. Crucially, DACSNPs-Col hydrogels exhibit more than 90% cell viability, less than 3% hemolysis and enhanced fibroblast adhesion due to optimized pore uniformity for 87.8 μm, demonstrating balanced biomechanical and biological functionality [68]. Compared with Genipin, DAS has a lower process cost, but the mechanical properties of cross-linked collagen produced by it are weaker, which is one of its main limitations. Dai *et al.* compared common cross-linking agents, Genipin, EDC/NHS and dual cross-linked (DC) materials to select the best cross-linking mechanism. DC materials present the highest slopes of the stress–strain curves, the highest compression modulus and storage modulus. These data indicated that the DC material possesses strong rigidity, good stability and superior mechanical properties [69].

Chemical crosslinking, the core strategy for the functional modification of collagen materials, constructs a stable polymer network by covalently modifying carboxyl/amino functional groups, thereby significantly improving the mechanical strength and thermal stability of the material. However, currently, there is no ideal chemical crosslinking method. The future development direction needs to balance crosslinking efficiency, biological safety and cost control and promote the collaborative innovation of natural/synthetic crosslinking agents to meet the needs of diverse clinical scenarios.

#### Physical crosslinking

Physical crosslinking strategies for collagen include ultraviolet (UV) irradiation and dehydrothermal treatment (DHT) [70]. As a nontoxic alternative to chemical approaches, physical crosslinking avoids cytotoxic reagents but often yields gels with suboptimal mechanical properties [71]. UV irradiation generates reactive free radicals that facilitate intrafibrillar and extrafibrillar covalent bond formation between aromatic amino acid residues. However, prolonged UV exposure leads to progressive denaturation of collagen, which undermines its structural integrity. Collagen gelation under 254 nm UV irradiation was completed within 15 min, producing materials with enhanced enzymatic resistance [72]. DHT induces collagen gelation via water removal under elevated temperatures and vacuum, enabling amide bond formation between carboxyl and amine groups [73]. Industrial protocols typically apply a 0.05 mbar vacuum at 40°C to facilitate water removal while mitigating collagen denaturation. Subsequent thermal treatment at temperatures exceeding 100°C promotes advanced gelation. Notably, gelation and denaturation processes may occur concurrently. Elevated temperatures may induce collagen denaturation, resulting in suboptimal tissue architecture [74]. Studies have indicated that excessively high temperatures or prolonged treatment duration impede collagen optimization. Within a specific range, the crosslinking density increased with temperature and time. However, excessive temperature and prolonged durations damage the triple-helical structure of collagen, leading to a significant reduction in its mechanical properties. Collagen films treated at 125°C for one day or 105°C for three days exhibit superior mechanical properties [59, 75]. Although the UV method is more efficient and faster than DHT treatment, DHT-cross-linked collagen exhibits higher sensitivity to trypsin and greater resistance to degradation, showing a lower dissolution rate of collagen fibers in collagenase solution. Additionally, the DHT method is more suitable for processing thicker materials than the UV method [76].

Physical crosslinking facilitates the construction of a collagen network through UV and DHT irradiation, thereby avoiding the toxicity risk of chemical reagents. Although the mechanical properties of physical crosslinking are weaker than those of chemical methods, they have become the preferred strategy for skin repair and bone filling materials because of their advantages, such as the absence of toxic residues and high structural controllability in tissue engineering. In the future, it will be necessary to overcome this performance bottleneck through parameter optimization and cooperation with chemical and biological crosslinking.

#### Enzymatic crosslinking

The enzymatic crosslinking method for collagen involves biocatalytic reactions to achieve intermolecular crosslinking. Collagen crosslinking and stability *in vivo* are primarily governed by enzymatic reactions. Enzymatic crosslinking is also effective under *in vitro* conditions. Enzymatic crosslinking is favored over physical and chemical methods because of its excellent biocompatibility, mild reaction conditions, lack of by-products, high specificity and high catalytic efficiency and yield. Furthermore, enzymatic reactions generally occur under normal temperatures and pressures, eliminating the need for extreme conditions such as high temperatures and pressures. This advantage helps preserve the native structure and bioactivity of the collagen. Enzymatic crosslinking employs lysyl oxidase (LOX), microbial transglutaminase (MTG) and horseradish peroxidase (HRP). These enzymes catalyze amino group modifications and fibrous bond formation, demonstrating high catalytic efficiency and strong environmental responsiveness [77, 78]. Under physiological conditions, collagen undergoes enzymatic post-translational modifications to maintain stability, elasticity and biological activity. Among the enzymes used to enhance collagen mechanical strength, MTG is currently one of the most well characterized [79]. MTG primarily catalyzes the acyl transfer reaction between the γ-carboxamide group (acyl donor) of glutamine residues and ε-amino group (acyl acceptor) of lysine residues in collagen peptide chains, forming intermolecular and intramolecular ε-(γ-glutamyl)-lysine covalent bonds. This crosslinking approach maintains the native triple-helical conformation of collagen while improving its mechanical properties and thermal stability [80]. MTG crosslinking increases compressive strength of collagen-hydroxyapatite (HAP) composites from 1.10 MPa to 4.54 MPa, approaching the mechanical properties of human cancellous bone. Furthermore, as demonstrated in bilayer osteochondral scaffolds, MTG enables fusion of cartilage and subchondral bone layers without structural gaps, facilitating spatially organized tissue regeneration [81]. HRP is a commercially available, widely used, plant-derived peroxidase. HRP catalyzes phenol-rich polymers using H_2_O_2_ as an oxidant, effectively improving the mechanical properties of collagen hydrogels. For Col II, HRP crosslinking enables covalent functionalization via tyramine grafting (Col II-Tyr) while preserving its triple-helical conformation. In hybrid HA-Tyr/Col II-Tyr hydrogels, HRP tuning achieves rapid gelation in 25–170 s with higher HA-Tyr content increasing storage modulus up to 4510 Pa and reducing hydrogel shrinkage. Critically, HRP-crosslinked Col II-Tyr enhances chondrogenic differentiation of encapsulated mesenchymal stromal cells (hBM-MSCs), upregulating expression of genes such as *COL2A1*, *ACAN* and *SOX9*, while promoting glycosaminoglycan deposition as well [82].

Compared with chemical or physical methods, enzymatic crosslinking is completed at mild temperatures and atmospheric pressure, without toxic by-products, and with high reaction efficiency. It is particularly suitable for sensitive tissue-engineering scenarios. Their environmental response characteristics provide a new dimension in the design of intelligent biomaterials. However, enzyme activity is easily affected by the environment. The high cost and stability of large-scale production remain as major challenges. In the future, through enzyme immobilization, directed evolution or a collaborative strategy with synthetic crosslinkers, the application boundaries in the fields of regenerative medicine and drug delivery can be expanded.

### Blending

The fabrication of collagen-based biomaterials often involves blending, which is another common approach to enhance collagen-based biomaterials. Collagen-based materials can be engineered into various physical forms, including scaffolds, hydrogel films, microspheres, microparticles, nanoparticles and coatings, during the fabrication process, where blending strategies are simultaneously achieved. Blending strategies commonly involve the addition of growth factors or drugs to augment the functionality. However, this approach is largely contingent on specific application needs, and thus, falls outside the scope of this chapter. The following section discusses blending strategies used in the fabrication of collagen-based biomaterials.

#### Blending during fabrication of gelation

**Figure 2.**
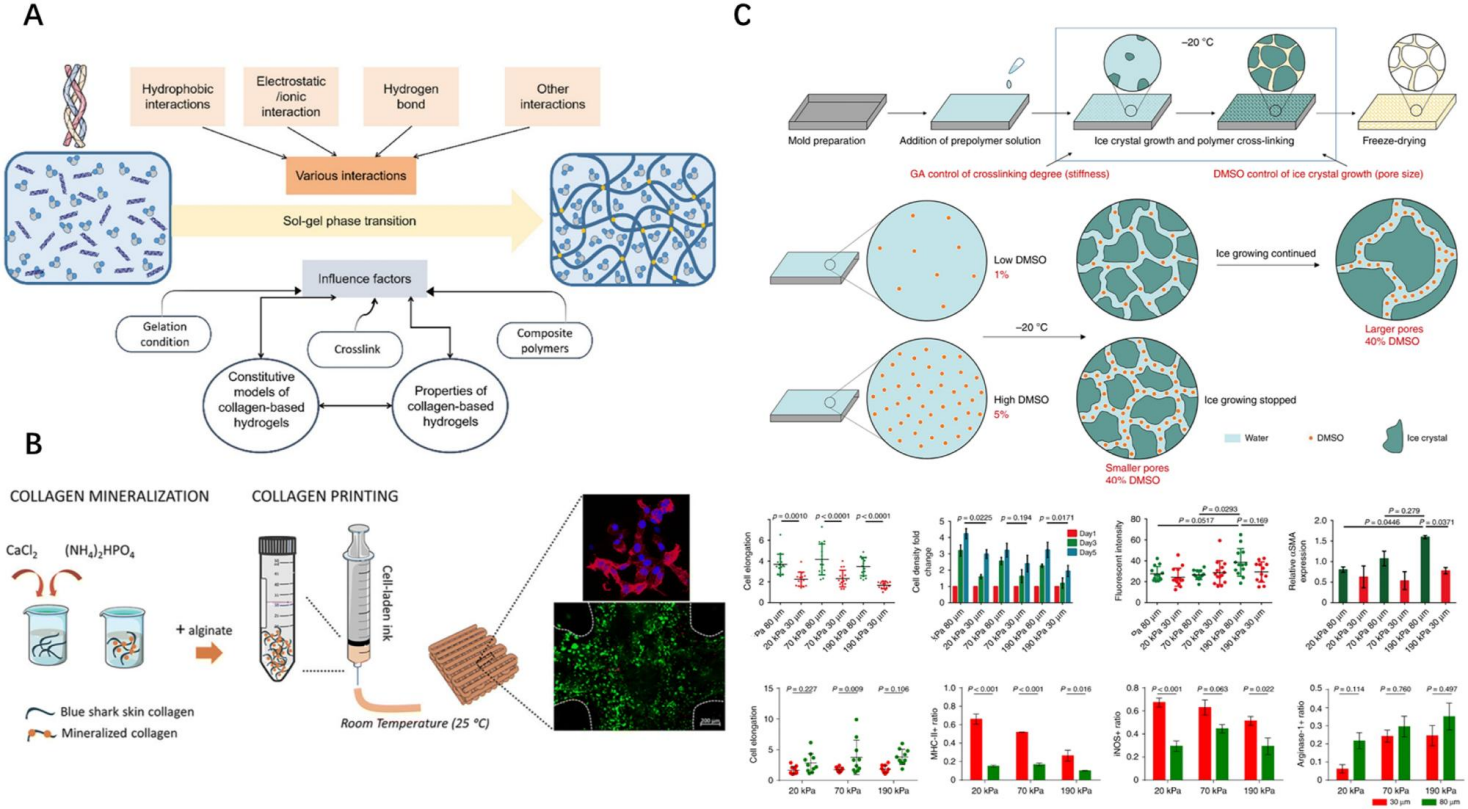
Blending strategies employed during the production of collagen-based biomaterials. (**A**) The gelation mechanism from collagen hydrogels. Reproduced from Ref. [25] with permission of Elsevier, © 2025. (**B**) 3D printing techniques based on collagen-based inks and 3D printed hydrogels made from shark-skin collagen, which are biomimetically mineralized and include living cells, are used for engineering hard tissues. Reproduced from Ref. [84] with permission of Royal Society of Chemistry, © 2025. (**C**) Precise control of scaffold pores sizes by cryoprotectant-regulated ice crystal growth leads to independent control of scaffold stiffness and pore size. Fibroblasts respond to the stiffness and pore size of 3D porous scaffolds and BMDM phenotypes can be modulated by pore size and stiffness of the scaffolds. Reproduced from Ref. [85] with permission of Springer Nature, © 2019.

The traditional synthesis of collagen hydrogels involves two steps: (i) neutralizing acidic monomers with phosphate-buffered saline (PBS) and NaOH solution and (ii) self-assembly by warming the solution to physiological temperature [83]. During the process, the gelation mechanism of collagen hydrogels involves two steps. First, crosslinking occurs between collagen fibers through intermolecular interactions to form an initial network, and then, free collagen molecules are rapidly incorporated into the 3D network during self-assembly. Factors such as temperature, collagen concentration and pH influence sol-gel phase transition, thereby altering the mechanical and rheological properties of the hydrogel (Figure 2A) [25]. The incorporation of inorganic and organic polymers into collagen networks can tailor the material properties of hydrogels. Widely used polymeric additives include alginate, chitosan and cellulose [83]. For instance, Li *et al.* fabricated a sulfated bacterial cellulose modified with sulfate composited with gelatin to improve the mechanical properties of gelatin scaffolds. They found that gelatin scaffold has only a compression modulus of 6.577 kPa, while the composites mixed with celluloses have increasing compression modulus of 37.59, 39.71, 41.78 and 45.18 kPa as the degree of substitution increases [86]. Distinct crosslinking strategies and optimized parameters were selected based on the incorporated components to engineer hydrogels with specific physical characteristics.

#### Blending during fabrication of scaffold

Fabricated from collagen-based matrices, these biomimetic scaffolds possess 3D porous architectures that maintain structural integrity under physiological loads, thereby matching implantation site requirements [8]. The regenerative capacity of a scaffold depends on key determinants, including mechanical strength, interconnected porosity and bioactive surface properties, which collectively govern tissue regeneration processes. Collagen scaffolds with additive components are more viable than pure collagen materials for tissue engineering and regeneration in sports. They facilitate regeneration and repair of bones, cartilage, tendons and ligaments. Hence, we introduced several blending techniques for the fabrication of collagen scaffolds.

Sufficient mechanical strength is essential for scaffolds to withstand mechanical load after implantation. The use of collagen blends to fabricate composite fibrous matrices is a rational approach for engineering new materials with the desired properties [87]. Collagen scaffolds comprise organic bioactive molecules blended with synthetic polymers, including poly(lactide-co-glycolic) acid (PLGA)/collagen, polycaprolactone (PCL)/collagen, poly(l-lactide) (PLLA)/collagen and polydioxanone (PDO)/collagen [88–90]. Established scaffold fabrication techniques include solvent casting/particle leaching, freeze-drying, electrospinning and additive manufacturing methods such as rapid prototyping and 3D printing. Freeze-drying constructs porous architectures by vitrifying aqueous collagen solutions to generate ice crystal networks, followed by sublimation under reduced pressure [91]. Freeze-drying is the most widely adopted method, as it maintains the native structure and bioactivity of collagen while enabling the incorporation of active ingredients. However, it lacks efficiency in terms of resource utilization, cost-effectiveness and time management. In addition to superior mechanical properties, coordinated regulation of porosity, pore size and stiffness in collagen scaffolds is a critical determinant of their functional performance [8, 85, 92]. For example, collagen scaffolds with varying pore sizes elicit different biological responses during osteochondral and tendon tissue regeneration [92–94]. Numerous methods have been developed to regulate the porosity of the collagen scaffolds. During freeze-drying, pore size and connectivity can be readily adjusted using preformed ice particles [8]. Jiang *et al.* modulated cryopreservation gelation using cryoprotectants, enabling control over the porosity and stiffness of the gelatin scaffolds. They found that fibroblast and macrophage phenotypes respond to scaffold physical properties, demonstrating that stiffer scaffolds with larger pores favor reduced inflammatory responses and enhanced tissue regeneration (Figure 2C) [85].

3D printing, an additive manufacturing technology based on layer-by-layer deposition, offers capabilities to develop complex scaffolds and organoids when using multiple cell types and biomaterials. As a 3D printing bioink, collagen can be blended with organic polymers or inorganic fillers to achieve the rheological properties, rapid gelation and structural stability required for 3D printing (Figure 2B) [83, 84]. Zhang *et al.* fabricated a 3D-printed scaffold using a photopolymerizable bioink composed of recombinant collagen, chitosan, Laponite-XLG and Kartogenin (KGN)-loaded nanoparticles. The scaffold demonstrated good cytocompatibility with human bone marrow mesenchymal stem cells (hBMSCs) and excellent antimicrobial properties. The continuous release of KGN induces hBMSCs differentiation into chondrocytes, offering new therapeutic opportunities for cartilage injury-related diseases [95].

Electrospinning has emerged as a novel technique for the fabrication of collagen nanofibers. The principle involves forming and elongating polymer solution droplets into fibers via an electric field generated by a high-voltage power source to overcome surface tension. This method enables precise control of the arrangement and morphology of collagen fibers. The resulting electrospun collagen nanofibers integrate the structural and chemical properties of the nanofibers and collagen, exhibiting benefits such as a large specific surface area, high porosity, excellent biocompatibility and abundant reactive groups [96]. However, biopolymers suffer from rapid degradation and are highly soluble in aqueous environments. Thus, crosslinking to regulate their water solubility and blending with organic polymers during electrospinning can enhance their biological properties and mechanical strength [90].

### Alignment of collagen fibers

**Figure 3.**
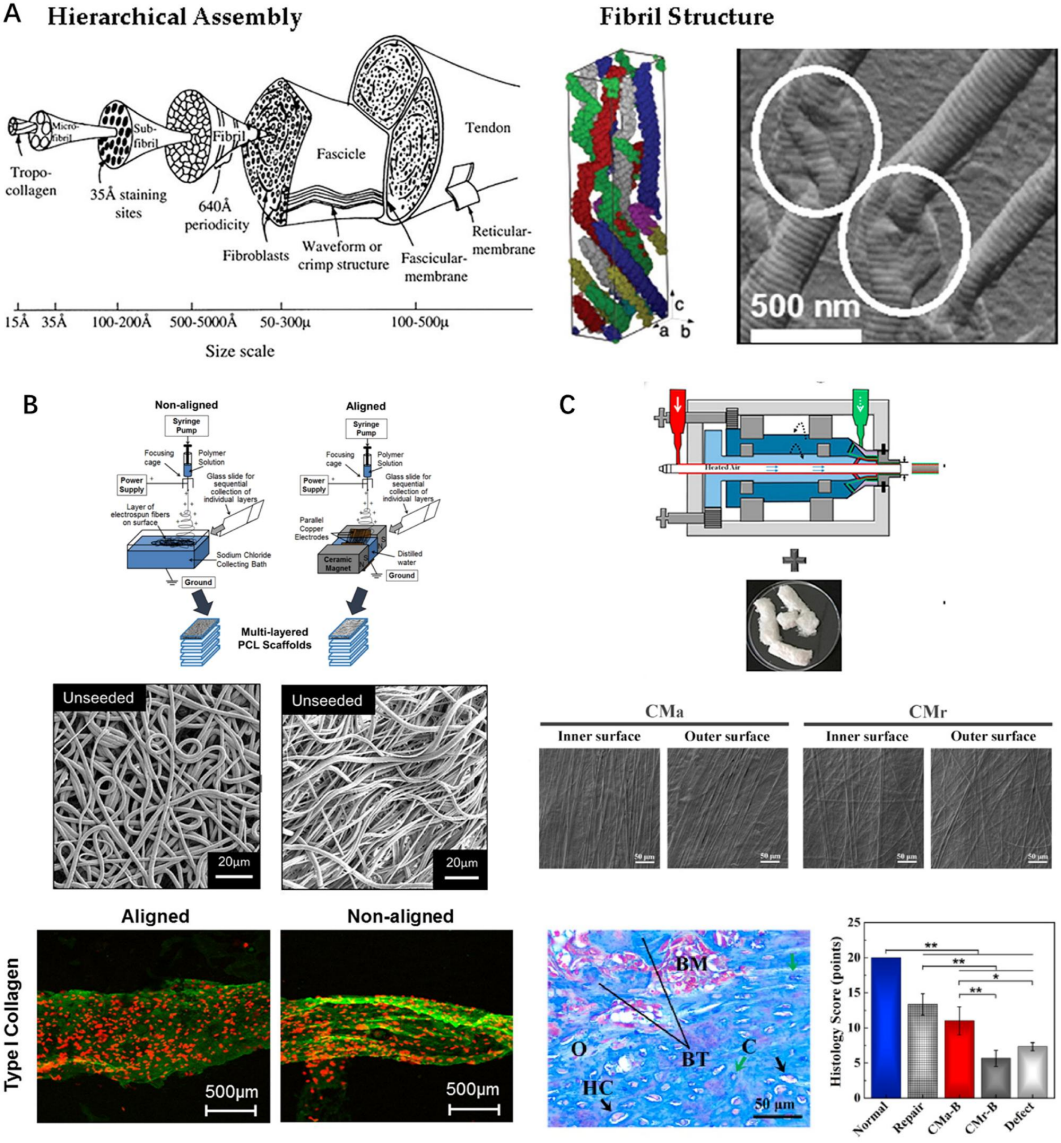
The alignment of collagen is of great significance for simulating the microstructural organization of tendons in a physiological state. (**A**) Schematic of the structure of human tendon and Col I fibril. Reproduced from Ref. [101] with permission of Elsevier, © 2011. (**B**) Electrospinning apparatus for nonaligned and aligned electrospun scaffolds. Reproduced from Ref. [102] with permission of Elsevier, © 2015. (**C**) The CRE technology for fabricating an aligned collagen membrane from insoluble collagens (CMa) which can produce a comparable healing quality to the autogenous tendon. Reproduced from Ref. [103] with permission of Elsevier, © 2019.

While section Blending addressed the optimization of scaffold composition through blending, achieving functional tendon mimics requires not only appropriate material selection but also precise control over the microstructural architecture of collagen. Kato *et al.* found that a homogenized insoluble collagen gel could be extruded as a fiber by utilizing fibrillogenesis to fabricate pseudo-native structures and reported the first aligned collagen fiber scaffold [97]. Since then, the alignment of fibrous constructs, on both the nanoscales and microscales, has been shown in numerous studies to direct the axial alignment of tissue synthesis and is another critical strategy for mimicking the microstructural organization of tendon [98–100]. Optimizing the fiber alignment process has critical scientific and practical implications (Figure 3A) [101]. Currently, multiple techniques exist for fabricating highly aligned collagen fibers, such as electrospinning, 3D printing, photolithography, microfluidic shear flow and magnetic patterning [104–108]. Orr *et al.* fabricated a multilayer fiber-aligned rotator cuff reinforcement patch via electrospinning, and seeded it with human adipose-derived stem cells. On Day 28, cell infiltration, ECM collagen deposition and tenomodulin expression were assessed. The results indicated that the performance parameters of the aligned scaffold group outperformed those of the nonaligned scaffold group, demonstrating that fiber axial alignment correlates with the enhanced mechanical properties of the scaffold (Figure 3B) [102]. However, these processing methods are exclusively applicable to soluble collagen forms. In contrast, insoluble collagen comprises naturally cross-linked fiber aggregates with biomimetic molecular arrangements. This structural organization endows it with self-assembly capabilities and mechanical properties that closely resemble those of the native collagenous ECM components. Yang *et al.* optimized the method for injecting insoluble collagen gels using counter-rotating extrusion (CRE) to prepare collagen fibers with diverse alignment orientations. CRE is a process that forces insoluble collagen protein dough through a circular mold with an outer cone and inner cone. The researchers loaded the prepared collagen dough into an extrusion device, controlled the fiber alignment by adjusting the rotating extruder cone speed and integrated chemical and thermal physical crosslinking of GA to fabricate collagen membranes with distinct orientations. Furthermore, *in vivo* experiments demonstrated healing quality comparable to that of an autogenous tendon (Figure 3C) [103].

### Decellularization

**Figure 4.**
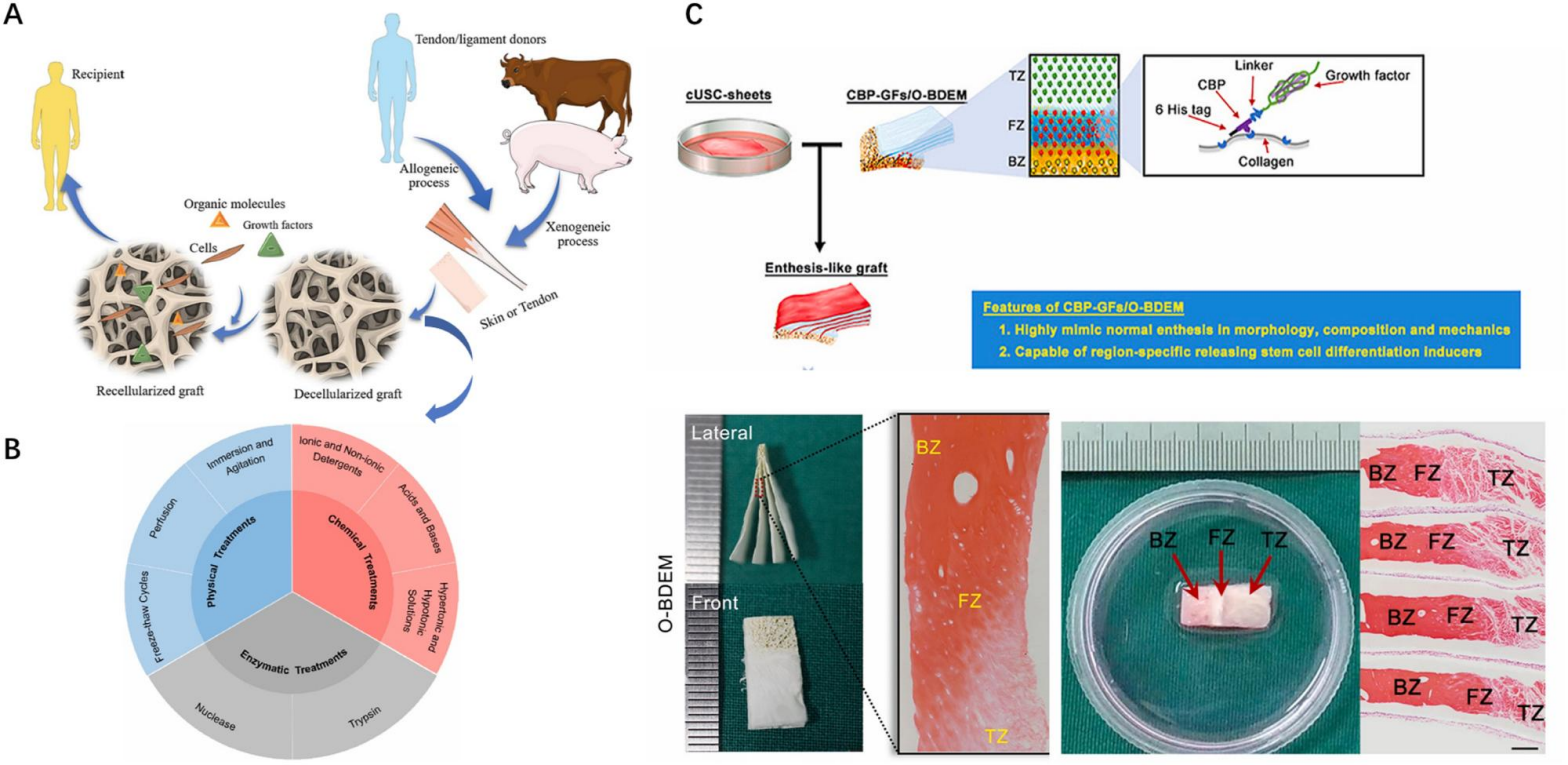
Technique of decellularization methods. (**A**) Allogeneic and xenogeneic process and transplantation. Reproduced from Ref. [110] with permission of Elsevier, © 2024. (**B**) Decellularization methods: physical treatments, chemical treatments and enzymatic treatments. The typical processes for each treatment are cataloged. Reproduced from Ref. [111] with permission of Elsevier, © 2022. (**C**) An approach to fabricate highly biomimetic scaffold capable of zone-specifically releasing stem cell differentiation inducers reproduced from Ref. [112] with permission of Elsevier, © 2022. Abbreviations: TZ, tendinous zone; FZ, fibrocartilaginous zone; BZ, bony zone.

Decellularization is another key tissue-engineering technique for enhancing the properties of collagen-based biomaterials. Decellularized extracellular matrix (dECM) refers to the native ECM structure isolated from allogeneic or xenogeneic tissues/organs via physical, chemical or enzymatic decellularization. In contrast, collagen scaffolds are typically fabricated from purified collagen, derived from natural sources or produced through recombinant technology. Key advantages of dECM include its high tissue-specificity, intricate bioactive components and native 3D architecture [109]. dECM-based biomaterials can be integrated with bioactive substances or cells, offering substantial advantages and promising applications in the treatment of musculoskeletal disorders, particularly tendon and cartilage repair [15]. This complex composition confers superior bioactivity and cell-instructive signals, enhancing cellular adhesion, proliferation, differentiation and tissue-specific regeneration. dECM scaffolds for tendon repair are predominantly derived from mammalian sources, including the porcine small intestinal submucosa, human dermis and porcine dermis. These scaffolds are frequently functionalized with structural proteins, signaling growth factors and progenitor cells to augment their bioactivity (Figure 4A). The tissue-inductive capacity of dECM scaffolds exhibited significant source-dependent heterogeneity. Tendon-derived dECM scaffolds preferentially induce tenogenic differentiation of tendon stem/progenitor cells (TSPCs) through mechano-transduction pathways, while simultaneously suppressing osteogenic differentiation via BMP/Smad signaling inhibition. Consequently, the native tendon–bone interface dECM demonstrates enhanced regenerative specificity for reconstructing the fibrocartilaginous transition zone in enthesis repair [110].

Decellularization techniques have reached maturity, encompassing (i) physical treatments such as ultrasonication, freeze-thaw cycling and agitation; (ii) chemical treatments using detergents, bases, acids, hypotonic/hypertonic solutions, alcohol and other solvents to disrupt cell membranes and junctions; and (iii) biological treatments involving trypsin and nuclease (Figure 4B) [113, 114]. The selection of decellularization techniques primarily depends on factors such as cell density, matrix thickness and tissue source in practical production, with each technique having distinct advantages and limitations [115].

Current decellularization methodologies require refinement in two critical aspects: complete removal of cellular components from large-scale ECM and preservation of biomechanical functionality during scaffold postprocessing. Chen *et al.* developed a protocol to address issues associated with conventional decellularization, such as incomplete chondrocyte removal and matrix ultrastructural damage during osteochondral interface preservation. They controlled partial decalcification of native tendon–bone specimens, and then, sectioned them into 250-μm-thick lamellar constructs along the collagen fiber direction. Decellularization via a vacuum aspiration device (VAD), which creates hydrodynamic shear forces, produced an osteoconductive book-shaped dECM (O—BDEM) with a preserved fibrocartilage transition zone (Figure 4C). *In vitro*, the scaffold effectively induced human urine-derived stem cells to differentiate into osteogenic, chondrogenic and tenogenic lineages, showing excellent biological activity.

Decellularized materials are being utilized in exciting new fields, such as nanofibers, hydrogels and bioinks, for bioprinting and disease modeling to investigate new therapies and significant progress has been made in research and development. In general, scaffolds based on decellularized matrices show tremendous potential for tendon and ligament regeneration. In the future, in addition to optimizing the decellularization process, developing ideal bioactive payloads and focusing on the immune modulation process during the regeneration of decellularized tissues will be the primary direction for the advancement of decellularization technology.

## Application in tissue engineering in sports medicine

With advancements in research and development processes and *in vitro* and *in vivo* experiments, the application of collagen in the treatment of sports injuries has increased annually. However, there is currently no research analyzing the status of collagen-based biomaterials in sports medicine and the overall information is scattered and fragmented. At the beginning of this section, we compiled clinical information from three platforms: ClinicalTrials.gov, trialsearch.who.int and www.chictr.org.cn. The search strategy was as follows: On the U.S. clinical trials registration platform, we limited the conditions to ‘condition or disease’ including cartilage defect, cartilage degeneration, cartilage injury, cartilage damage, cuff-tear arthropathy, shoulder pain, rotator cuff tears, Achilles tendon rupture, Achilles tendinopathy, bone loss, bone defect, ligament injury, ligament rupture, ligament tear and ligament knee injury. For ‘other terms’, we used ‘collagen’. To ensure comprehensiveness of the relevant research, we did not filter out information based on age, study start date, study phase, study type, study results or study staging. On trialsearch.who.int and www.chictr.org.cn, we directly searched for the keyword ‘collagen’ and subsequently screened the search results based on conditions that fall within the scope of sports medicine diagnosis and treatment. The inclusion and exclusion criteria were the same as those previously described.

**Figure 5.**
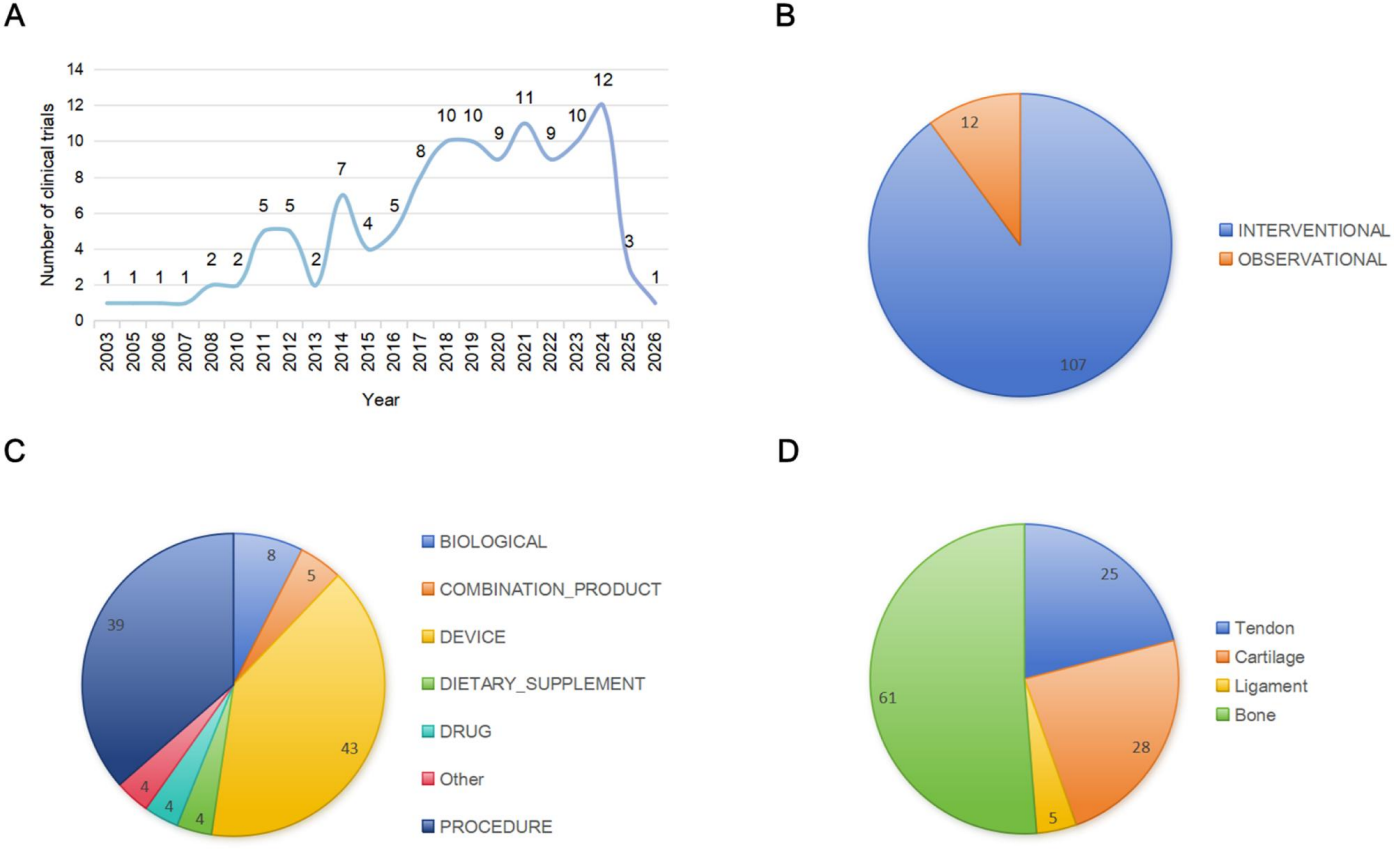
Current collagen related materials clinical studies based on ClinicalTrials.gov. (**A**) Year distribution of clinical studies on collagen. (**B**) Distribution map of clinical research type. (**C**) Distribution map of intervention type in interventional researches. (**D**) Composition of the types of tissue treated in clinical research. Date of research: 23 April 2025.

As of 23 April 2025, 119 clinical studies were retrieved from the three aforementioned platforms with the earliest registration date being 2003. Since 2015, an overall upward trend has been observed (Figure 5A). Regarding the type of studies, there were 107 interventional studies (89.92%) and 12 observational studies (10.08%) (Figure 5B). Among the interventional study types (Figure 5C), collagen was predominantly used in DEVICE (*n* = 43) and PROCEDURE (*n* = 39), accounting for 40.19% and 36.45%, respectively. BIOLOGICAL was the third most common intervention (*n* = 8, 7.48%), including injectable collagen, cell implantation therapy and other treatment methods. When grouping all included clinical studies based on tissue distribution (Figure 5D), collagen membrane-induced bone tissue regeneration accounted for the largest proportion (*n* = 61; 51.26%). Then collagen scaffold for cartilage repair turned out to be the second most abundant component (*n* = 28; 23.53%). And there were 25 clinical studies on tendon repair of the rotator cuff and Achilles tendon, accounting for 21.0%, with the treatment of rotator cuff tears comprising the majority (*n* = 21). However, few studies have been conducted on ligament regeneration therapy (*n* = 5). It is worth noting that among the treatments for collagen-induced bone tissue regeneration, clinical studies conducted in dentistry accounted for the majority. Although these cases may not be the focus of sports medicine, the clinical studies on bone tissue conducted in dentistry are relevant to the development of sports medicine. The role of collagen in regeneration and repair of different tissues is described in detail in the following sections.

### Tendon repair

According to our research presented above, collagen-based materials utilized in tendon repair take an important part in sports medicine with the focus on the rotator cuff and Achilles tendon. Tenocytes in tendons synthesize and support the maintenance of the ECM, where collagen (primarily Col I), accounting for 60–80% of the dry weight, serves as the main component responsible for the tensile strength of the ECM. Collagen molecules form a hierarchical structure consisting of tropocollagen (300 nm in length and 1.5 nm in diameter), microfibrils (composed of five collagen molecules), fibrils (with a diameter ranging from 50 to 200 nm), fibers (diameter of 3–7 µm) and fascicles (several micrometers in diameter), all arranged in parallel along the load-bearing axis [98, 99]. Given these characteristics, collagen, particularly Col I, exhibits promising potential for mimicking and repairing tendons [116].

#### Rotator cuff

In the surgical treatment of rotator cuff tears (RCTs), collagen matrix patches have emerged as a significant approach for improving postoperative healing outcomes because of their ability to promote tissue regeneration and enhance repair strength. RCTs, one of the most common tendon injuries in the shoulder, account for approximately 40% of all shoulder pain cases, and are a prevalent cause of shoulder pain and dysfunction [117]. The size of RCTs, which exhibit varying clinical symptoms and surgical outcomes, should be considered during treatment [118–120]. RCTs can be categorized based on their size into small (<1 cm), medium (1–3 cm), large (3–5 cm) and massive rotator cuff tears (MRCT). An acellular collagen matrix patch augmentation (ACMPA) is widely utilized for the enhanced repair of RCTs. Meta-analyses demonstrated that acellular collagen matrices significantly reduced re-tear rates (*P* = 0.0006) and improved functional outcomes (American Shoulder and Elbow Surgeons Score + 5.60, *P* = 0.01) in arthroscopic rotator cuff repair (ARCR) for tears ≥3 cm, whereas tears measuring 1–2 cm demonstrate comparable healing rates without requiring augmentation [121, 122]. Cai *et al.* presented a randomized controlled study on ARCR augmented with a biological collagen graft and summarized its application in medium-to-large tears [123]. A prospective randomized controlled trial reported a 24-month follow-up on cases augmented with collagen matrix patches, indicating that degenerative small- and medium-sized RCTs also benefit from surgery, with better strength and functional recovery reported [124]. However, further research, particularly to determine the indications for augmentation, is crucial [122]. Additionally, investigations are needed to identify the optimal graft choice, including whether allografts or xenografts are preferred, and to determine the ideal thickness of acellular collagen matrix patches [125].

#### The Achilles tendon

The Achilles tendon, which is the thickest tendon in the human body, has a relatively high incidence of rupture. Inadequate treatment of Achilles tendon rupture can significantly reduce the heel-raising ability, alter gait patterns and severely affect daily life and work. The treatment approaches for Achilles tendon ruptures include surgical and nonsurgical strategies [126, 127]. Surgical treatment is associated with a higher risk of postoperative complications including infection, nerve injury and tissue adhesion. Managing Achilles tendon ruptures in elite athletes presents a unique challenge. Even with surgical intervention, sports requiring explosive plantar flexion are linked to a more pronounced performance decline [128]. Recently, *in vivo* studies have shown that collagen membranes or scaffolds can accelerate tendon repair and enhance the functional recovery of healed tendons [129–131]. Yang *et al.* demonstrated the feasibility of using CRE technology to fabricate biomimetic collagen scaffolds for tendon regeneration. They employed CRE to process insoluble collagen into collagen membranes with controlled fiber orientation for tendon regeneration [103]. Yuan *et al.* fabricated poly(L-lactic acid) (PLLA) fibers surface-coated with Col I and chondroitin sulfate (CS) using coaxial stable-jet electrospinning for tendon regeneration. The results support the view that highly aligned biomimicking fibers may serve as an efficient scaffolding system for functional tendon regeneration [129]. Further investigation to characterize tendon regeneration could focus on the balance of collagen scaffold or membrane in exogenous and endogenous tendon healing mechanism. Cao *et al.* developed electrospun dual-functional nanofibers with surface robust super lubricated performance to regulate healing balance by reducing exogenous adhesion and promoting endogenous healing. It is conducted that the healing process could be improved by inhibiting friction-induced exogenous adhesion, which could be utilized in fabrication of collagen-based biomaterials for tendon regeneration [132].

As for clinical application, bio-inductive collagen scaffolds have been utilized in treatment for insertional Achilles tendinopathy (IAT) and Haglund deformity (HD) [133]. Genuth *et al.* conducted a retrospective case series reporting that collagen scaffolds appear to enhance patients’ outcomes diagnosed with IAT and HD without increasing complication rates. In an average follow-up duration of ten months, the average value of American Orthopedic Foot and Ankle Society improved from 62.3–89.6 at six months postsurgery (*P* < 0.05). Complete healing was proven by MRI at six months postoperation and assessment of preoperative function regaining in averaging nine months after surgery [134]. But future research is warranted to more comprehensively assess the benefits.

Injuries or degeneration of rotator cuff and Achilles tendon are common in sports medicine, and the tendons of these two regions require high mechanical strength. Collagen-based materials exhibit excellent biocompatibility and tissue-inductive properties coupled with adequate mechanical strength and the ability to mimic the arrangement of tendon tissue. In addition, with the hot topic in the development of exoskeleton rehabilitation system with different strategies including personalized attachment point optimization method and flexible assistance, more sophisticated research for tendon regeneration could focus on the combination of developing bioactive materials and providing acute rehabilitation model [135, 136]. Consequently, although the mechanical properties of pure collagen materials may not fully meet these requirements, integrated bio-based materials that utilize collagen as a foundation represent a highly promising avenue in the field of tendon regeneration.

### Ligament repair

Collagen has significant application value in ligament repair and reconstruction, owing to its excellent tissue compatibility and osteogenic activity. However, as shown in Figure 5, currently only a few clinical studies have been conducted on ligament regeneration therapy. Collagen based materials have been utilized mainly in preclinical studies or play their repairing function as auxiliary components for graft or artificial ligament. Constructing composite scaffolds or injectable products with collagen optimizes the graft–bone interface microenvironment to promote early postoperative functional recovery. This approach offers innovative solutions to enhance the long-term stability of reconstructive surgery.

The anterior cruciate ligament (ACL) is essential for knee joint stability and movement, making it susceptible to injury. ACL injuries not only cause knee instability but also increase the joint’s susceptibility to subsequent injuries and long-term degenerative changes. Owing to the poor regenerative capacity of the ACL after injury, autologous grafting, allografting or artificial ligaments are commonly employed for ACL reconstruction. Artificial ligaments, constructed using high-strength synthetic materials, have been engineered for ligament repair and reconstruction. Artificial ligaments generally display superior mechanical strength compared to autologous and allograft tissues. Implanting synthetic materials eliminates the ligamentization process, thereby preventing a decline in the graft mechanical strength resulting from tissue necrosis, vascularization and remodeling [137]. The ligament–bone interface, characterized by a complex arrangement of collagen, minerals and proteoglycans in a layered gradient structure, presents a significant challenge in ACL reconstruction: at the graft–bone interface, scar tissue formation frequently impedes the recovery process [138].

Combining collagen with other extracellular matrices and artificial ligaments potentially enhances the osteoinductivity of the graft tissues and promotes postoperative recovery. Yu *et al.* developed a multilayered nanofiber-reinforced 3D scaffold-integrated decellularized tendon for ACL reconstructions. This scaffold features a unique layered structure consisting of a decellularized tendon core, an intermediate layer of polyurethane/type I collagen (PU/Col I) yarn, and an outer layer of a poly(L-lactic acid)/bioactive glass (PLLA/BG) nanofiber membrane. *In vivo* rabbit ACL reconstruction models demonstrated that the composite group exhibited superior osteogenic performance and significantly higher ultimate stress. The central PU/Col I yarn layer is critical for promoting the tenogenic differentiation of tendon-derived stem cells and upregulating the expression of key tendon-specific factors [138]. Jiang *et al.* additionally addressed potential healing challenges at the bone-tendon interface. They engineered an injectable HAP/Col I paste to create an optimal local microenvironment at the tendon–bone interface following autologous ACL reconstruction. Validation was performed at the biomechanical and histological levels of the tendon–bone interface in a canine ACL reconstruction model [139]. In clinical practice, Götschi *et al.* utilized an osteoconductive scaffold (OCS) composed of a natural mineral matrix of bovine origin, reinforced with biodegradable synthetic polymers and natural collagen derivatives, for ACL reconstruction. They placed the scaffold at the femoral tunnel aperture and conducted a 2-year follow-up study. The outcome showed that there was no significant decrease in bone tunnel volume in the intervention group with OCS compared to the control group, and the procedure was free from additional adverse results [140]. Anz *et al.* attached an amnion collagen matrix to a tendon graft and injected bone marrow aspirate concentrate (BMAC) to overcome the lack of synovial lining after ACL reconstruction. Specifically, the intervention group underwent standard ACL surgery with three additional steps: (i) the bone marrow was harvested and concentrated to create the BMAC; (ii) the ACL graft was wrapped with an amnion collagen matrix; and (iii) after graft implantation, the joint was dried and the BMAC was injected under the amnion collagen matrix. This study showed that wrapping a graft with an amniotic collagen matrix and injecting BMAC appears safe. However, MRI T2 values and graft volume of the augmented ACL graft were not significantly different from those of the controls, suggesting that the intervention did not result in improved graft maturation [141]. These findings indicate that enhancing the inductive properties of grafts promotes early recovery, and that long-term follow-up is required to assess graft performance.

Recent years, the utilization of autologous ACL for *in situ* suturing and repair turns to be a promising treatment strategy for acute ACL rupture [142]. The procedure is achieved by the Bridge-Enhanced ACL Repair (BEAR) Implant. The U.S. Food and Drug Administration (FDA) granted the marketing authorization of BEAR Implant in December 2020. The BEAR does not require the use of harvested tendons for ACL repair and is the currently available alternative to traditional ACL reconstruction in the USA. The development of BEAR began with a biological scaffold constructed from collagen materials. Murray *et al.* fabricated a scaffold composed of collagen and platelet-rich plasma (PRP) for the repair of porcine ACL [143]. They found that the composite scaffold (COL/PRP) significantly increased the maximum load-bearing capacity of the ACL in a flexed knee position and promoted ACL repair. It was further validated by subsequent studies and clinical trials. Murray *et al.* conducted a 2-year follow-up randomized controlled trial including 100 patients with complete midsubstance ACL injuries who were randomly assigned to receive either BEAR or autograft ACLR. In total 96 returned patients, the BEAR group had a significantly higher mean hamstring muscle strength index than the ACLR group at two years, while their rate and outcome of a second ipsilateral ACL surgical procedure turned out to be similar [144]. To optimize the efficacy of BEAR implant, Sun *et al.* developed a composite scaffold comprising collagen (1%, w/v) and polyvinyl alcohol (5%, w/v) combined with PRP. The composite scaffold provides a protective barrier against synovial erosion for the ruptured ACL, while simultaneously facilitating tissue repair, thereby enhancing the efficacy of ACL repair techniques [145]. The results proposed composite scaffold holds great promise as a candidate for ACL healing.

Owing to the mechanical limitations of collagen fiber materials, it is difficult to construct pure collagen materials or ligament substitutes using collagen materials as the core. Leveraging collagen’s tissue compatibility and osteogenic properties, collagen scaffolds and injectable collagen-based biomaterials enhance the microenvironment at the ligament–bone reconstruction interface. These scaffolds promote postoperative rehabilitation and optimize the long-term functional outcomes after ligament reconstruction.

### Cartilage repair

After COLTRIX CartiRegen being the first products approved by National Medical Products Administration in 2024 (Table 1), collagen-based biomaterials turned out to be a significant component for cartilage repair in terms of the present and the future with more and more related clinical trials as shown in Figure 5. As the principal functional component of the articular cartilage ECM, collagen demonstrates significant potential for cartilage repair owing to its synergistic biomechanical properties and bioactive characteristics. Mature articular cartilage contains various collagen molecules including Col II (accounting for more than 90%), type III collagen (10%), type IX collagen (1%), type XI collagen (3%) and type VI collagen (less than 1%). The structural versatility and high abundance of collagen render its fibrillar networks predominantly responsible for mechanical load-bearing in articular cartilage. Col I-based biomimetic scaffolds address the mechanical deficiencies of fibrocartilage generated by conventional repair methods and offer a superior alternative for articular lesion regeneration.

Unlike bone or other tissues, articular cartilage is avascular and has limited regenerative capacity after injury. Osteoarthritis progression involves cartilage degeneration, where sustained inflammation and osteoproliferative responses compromise the functional integrity of the femorotibial weight-bearing interface [146]. Current clinical interventions for repairing cartilage defects include microfracture (MF), chondroplasty and autologous chondrocyte implantation (ACI). However, MFs predominantly generate fibrocartilage with suboptimal biomechanical properties [147]. Chondroplasty is limited by the possibility of donor-site morbidity and lacks integration with the natural cartilage [148]. ACI demonstrates suboptimal efficacy for focal defects (<2 cm^2^), with persistent challenges including donor-site morbidity and monolayer-induced chondrocyte dedifferentiation [149]. These limitations have driven the development of cartilage tissue engineering (CTE) strategies [150–152]. The CTE encompasses three essential components: (i) implanted chondrogenic cells, (ii) biomechanically competent 3D scaffolds and (iii) modulation of bioactive signaling. Current clinical translation of CTE primarily involves matrix-induced autologous chondrocyte implantation (MACI) and autologous matrix-induced chondrogenesis (AMIC).

The clinical integration of MACI in contemporary orthopedic practice demonstrates the potential for structural restoration of cartilage defects. Similar to its predecessor, ACI and MACI involve two-stage surgical protocols. MACI’s key innovation employs a 3D scaffold architecture that prevents cellular leakage, while maintaining the chondrogenic phenotype through microenvironmental regulation [153]. Collagen-based scaffolds serve as pioneering matrices for MACI's initial clinical application of MACI [154, 155]. Although Col II predominates in native cartilage, pure Col II hydrogels demonstrate suboptimal mechanical strength, despite their chondrogenic potential [150, 156]. In contrast, Col I effectively supported articular chondrocyte (AC) and mesenchymal stem cell (MSC) viability, establishing it as a primary candidate for biomimetic scaffold fabrication. FDA-cleared Col I products, including NeoCART^®^, NovoCART^®^3D and CaReS^®^, have advanced through regulatory pathways [157–159].

Recent advances have augmented MF techniques with AMIC or matrix-associated BMS (M-BMS) by combining biological stimulation with scaffold-guided regeneration. The AMIC single-stage protocol (‘one-step method’) reduces the surgical burden compared with MACI, demonstrating accelerated rehabilitation timelines and improved patient compliance. Nevertheless, a comprehensive evaluation of the long-term clinical outcomes, cost-benefit ratios and procedure-related complications is imperative. Collagen scaffolds have demonstrated extensive clinical utility in cartilage regeneration, exemplified by commercially available biomedical products. In China, COLTRIX^®^ CartiRegen represents a pioneering registered collagen-based regenerative medical product for cartilage repair. This product is indicated for arthroscopic MF procedures targeting knee cartilage defects (2–8 cm^2^) with Outerbridge grade III/IV lesions (equivalent to ICRS III–IVa). Another clinically validated product, Chondro-Gide^®^, is specifically indicated for osteochondral lesions of the talus exceeding 1.0 cm^2^ [160]. Preclinical studies have demonstrated that its performance, biocompatibility are qualified and animal experiments have shown that it can promote cartilage regeneration. Clinical trials have shown that the Magnetic Resonance Observation of Cartilage Repair Tissue score of the test group was higher than that of the control group at 12 months. Offshore data for six years have shown that the relevant scores have been stably maintained. Adverse events were not clearly related to the device, and approval for registration is recommended [161]. Weigelt *et al.* conducted a long term clinical follow up that included 33 patients with osteochondral lesions of the medial talar dome who were retrospectively evaluated after open AMIC repair at a mean follow-up of 56 months. The trial demonstrated significant pain reduction and functional recovery of AMIC. Its mean visual analog scale score for pain significantly improved from 6.4 preoperatively to 1.4 at the latest follow-up. The mean AOFAS score for ankle function reached 93.0, indicating substantial recovery of daily activity function. Seventy-nine percent of patients returned to their previous sports levels with no cases required revision surgery for failed AMIC [162]. It is worth noting that, beyond application in knee cartilage repair, autologous nasal chondrocyte tissue-engineered cartilage (N-TEC) formed using the Chondro-Gide^®^ collagen membrane has proven to be an effective treatment for nasal septum perforation [163]. Subjective scoring improved in all patients in the 1-year post surgery follow up with no side effects such as SAR and no challenge in graft manipulation. These results are revealing that application of same products in different component with similar tissue distribution may accelerate the clinical translation.

### Bone repair

Collagen biomaterials exhibit exceptional biocompatibility, customizable mechanical characteristics and osteoinductive potential, making them promising candidates for bone regeneration and periodontal tissue engineering as shown in Figure 5D. Through engineered mineralized scaffolds, functional collagen matrices and bioactive factor delivery platforms, collagen-based biomaterials demonstrate (i) enhanced osseointegration kinetics, (ii) alveolar ridge preservation (ARP) capacity and (iii) dual soft tissue repair/regeneration potential, all critical for clinical translation.

Bone tissue, characterized by its highly vascularized nature, can spontaneously regenerate and repair damage. Despite advances in regenerative medicine, critical-sized bone defects pose persistent challenges in orthopedics, primarily owing to the compromised osteogenic potential in patients with severe trauma, skeletal pathologies or oncological conditions. Regarding treatment, autologous bone grafts, which remain the gold standard, are still constrained by donor-site morbidity and limited tissue availability [164, 165]. Consequently, the development of improved bone substitutes represents a critical research frontier in orthopedics. Among these, synthetic polymer-based hydrogels have shown promising results in preclinical studies, particularly in the field of bone tissue engineering (BTE). Given the hierarchical structure of bone-mineralized collagenous matrix, collagen-based composites have substantial potential for osteoregenerative applications [166]. Bai *et al.* integrated collagen type II with procyanidin (PC) onto an implant coating using a layer-by-layer technique. Osteogenesis-related genes were detected in co-cultured bone marrow mesenchymal stem cells (BMSCs), and an *in vivo* experiment was performed to reveal the effect of the newly designed material on the osteogenic differentiation of BMSCs. The results demonstrated that in BMSCs, PC/collagen multilayers accelerated proliferation and osteogenic differentiation through the Wnt/β-catenin signaling pathway, which may explain the improvement in the bone defect model of rabbit femurs [167].

Although biomaterials serve as the structural foundation for BTE scaffolds, their osteoinductive capacity is frequently augmented through structural or surface modifications. A particularly promising strategy involves collagen-functionalized polymer scaffolds that simultaneously enhance cellular compatibility and tailor the mechanical properties. The integration of bioactive additives into Col I-based composites results in synergistic improvements in mechanical integrity and bioactivity, thereby enhancing osteoregenerative efficacy and enabling drug delivery functionality [165, 168–170]. Composite scaffolds synthesized using Col I as the organic phase and solid silica particles (Sol-Si) or HAP as the mineral phase are common examples of such composites [171–173]. Zheng *et al.* developed mineralized collagen-based bone scaffolds (MC), porous MC (pMC) and compact MC (cMC) to construct a biphasic MC composite bone scaffold (bMC) for repairing large cranial bone defects in developing sheep. The pMC and cMC play different roles in bone regeneration, and the pMC is gradually replaced by regenerative bone tissues, whereas the cMC frame promotes new bone formation beneath the frame without obvious degradation, thus, providing appropriate mechanical protection and ensuring the structural integrity of the implant [174, 175]. Moreover, collagen-based scaffolds enable controlled release of recombinant human bone morphogenetic protein-2 (rhBMP-2), thereby augmenting osteoregenerative outcomes in preclinical models [176].

Bioactive glass (Bioglass), originally developed for bone grafting, has led to representative products such as Novabone^®^ for orthopedic use and Perio-Glass^®^ for maxillofacial surgery [177]. Collagen-bioactive glass (COL-BG) composites, predominantly consisting of silica (SiO_2_) and oxides such as sodium oxide (Na_2_O), calcium oxide (CaO) and phosphorus pentoxide (P_2_O_5_), have exhibited enhanced bioactivity in preclinical models [178].

Additionally, collagen matrices are frequently blended with natural or synthetic biopolymers to engineer grafts with optimized mechanical strength, biodegradability and osteoconductive properties [179–183]. These strategies involve simultaneous collagen self-assembly and mineralization initiation, where metal ions aggregate both intracellularly and on fibril surfaces, yielding biomimetic mineralization patterns analogous to native bone microarchitecture [184]. Despite preclinical advancements in collagen-based scaffold systems, translational research in the clinical setting remains underdeveloped. Wang *et al.* developed a bioactive composite bone cement for the treatment of osteoporotic vertebral compression fractures in which mineralized collagen (MC) was incorporated into poly(methyl methacrylate) (PMMA) bone cement (MC-PMMA) [185]. They compared the clinical effect of the composite with that of pure PMMA bone cement in a follow-up of two years. Both the Visual Analog Score and Oswestry Disability Index were significantly reduced when the MC-PMMA cement was used. These new bone cements have demonstrated superior long-term clinical effects. Further clinical trials have been conducted to evaluate their safety and efficacy. In addition to mineralized collagen, small intestinal submucosa incorporated into PMMA (mSIS-PMMA) also shows promising potential as an injectable biomaterial for orthopedic procedures that require bone augmentation [186].

Owing to their osteoinductive potential, collagen membranes have demonstrated clinical utility in dental applications, particularly in periodontal and maxillofacial surgeries. The alveolar bone serves as the foundation for teeth and plays a crucial role in maintaining chewing function. Over the past decade, collagen membrane-mediated guided bone regeneration (GBR) has emerged as a standard treatment for alveolar bone defects [187]. Collagen membranes contribute to osteogenic outcomes owing to their osteoconductive and biocompatible properties. However, their efficacy in augmenting bone regeneration within critical-sized defects, including periapical lesions remains suboptimal [188]. Clinical evidence indicates that collagen membrane therapy may correlate with increased cortical plate formation following endodontic surgery [189]. Liu *et al.* compared various regenerative medical techniques for the surgical treatment of persistent apical periodontitis, with and without the use of regenerative medical technology. Meta-analysis revealed that the use of collagen membranes or autologous platelet concentrates alone was associated with a trend toward better results (RR: 0.51; 95% CI: 0.20–1.25; *P* = 0.14) and (RR: 0.55; 95% CI: 0.18–1.71; *P* = 0.30), respectively. Only the combined use of collagen membranes and bovine-derived HAP showed significant improvement in results (RR: 0.35; 95% CI: 0.17–0.75) [190]. Collectively, collagen-based membranes exhibit rapid biodegradation kinetics and suboptimal dimensional stability within osteogenic niches. Premature membrane exposure following gingival dehiscence promotes premature degradation and compromises osteoregenerative outcomes. Collagen biodegradation kinetics and mechanical integrity can be optimized via crosslinking protocols and incorporation of bioactive agents.

ARP seeks to mitigate postextraction alveolar bone resorption. The aim of the ARP technique is to minimize resorption after tooth extraction. One of the most common strategies in clinical practice involves the use of a combination of graft materials covered with resorbable membranes [191]. Numerous clinical trials have employed collagen membranes in ARP procedures. A meta-analysis, which included 12 studies with 390 subjects, indicated that the use of bone substitute materials covered with collagen membranes achieved better ARP in both horizontal and vertical directions than spontaneous healing, although this trend was not statistically significant [192]. Additionally, compared to the use of collagen membranes alone, the addition of materials resembling allogeneic bone mineralization, composed of hyaluronic acid and beta-tricalcium phosphate, resulted in improved ARP, with fewer ridge alterations observed in both the vertical and horizontal directions [193].

### Translation potential

Clinical translation of collagen-based sports medicine products remains bifurcated. While devices addressing critical unmet needs achieve rapid commercialization through material innovation and regulatory fast-tracking, others, particularly multinational trials, face delays from evidence gaps or publication lags. Future translation requires: (i) leveraging expedited regulatory pathways for orphan indications and (ii) adopting pragmatic trial designs to accelerate real-world evidence generation.

Regulatory pathways and approved products mainly consist of two ways, including Innovative Medical Device Pathway in China and International Collaborative Trials. Excellent and innovative products could be approved by pathways with evident preclinical and clinical trials. For instance, the COLTRIX^®^ CartiRegen collagen scaffold has been previously based on robust preclinical research. Then, the applicant selected a clinical trial pathway for clinical evaluation to pursue product approval. They conducted a prospective, multicenter, noninferiority, single-blind, randomized controlled trial. A total of 105 subjects were enrolled and randomly assigned in a 1:1 ratio, with 52 subjects in the test group and 53 in the control group. The control group received MF surgery. All enrolled patients had femoral condyle cartilage defects in the knee joint. The applicant also provided overseas clinical data with an observation period of up to six years for the product under review. Follow-up assessments of IKDC scores, Lysholm scores and KOOS scores demonstrated that these scores progressively increased during the first two years and remained stable through the 6-year follow-up period [16]. In China, Bangsai Technology Co., Ltd’s Tendon Repair Matrix turned out to be the first domestic rotator cuff patch which exemplifies accelerated translation. Its approval leveraged ultra-high purity collagen technology, biomimetic 3D porous microstructure, tissue-inducible regeneration design philosophy with surgical compatibility minimizing learning curves.

In current regulatory pathways, one of the available strategies and current tendency is the application of registered collagen-based biomaterials cross-used in different components of human body with similar tissue distribution. For instance, a silk fibroin membrane succeeded in skin repair in 71-patient RCT [194]. For its utilization in tendon repair, the clinical trials for re-validation have been conducted [195]. As the COLTRIX^®^ CartiRegen has been approved in repair of cartilage, researchers in hospital of Obstetrics and Gynecology affiliated to Fudan University are conducting a clinical study of the safety and efficacy of COLTRIX^®^ CartiRegen collagen repair scaffold for the prevention of recurrent intrauterine adhesion [195]. In conclusion, the realization of promising translation potential in biomaterials require more innovative clinical trials and regulatory pathways.

## Perspectives

In the field of sports medicine, collagen-based biomaterials, owing to their unique tissue characteristics and ease of accessibility, have been successfully applied in the repair of tendon, bone and cartilage injuries, thereby achieving commercialization. These materials exhibit significant advantages in specific conditions including RCTs and cartilage defects. Collagen-based biomaterials offer innovative solutions for clinical treatment by providing mechanical support, promoting joint mobility and accelerating tissue healing.

Tissue-engineered collagen scaffolds are emerging as a key research focus owing to the increasing demands of patients and clinicians for improved reconstruction outcomes, particularly in the regeneration of articular cartilage. Incorporating growth factors or seed cells into bioactive repair systems can improve the microenvironment for tissue regeneration and vascularization for the treatment of cartilage loss and bone defects. Collagen-mediated osteogenesis represents a critical convergence point between musculoskeletal regeneration and dental rehabilitation, with shared molecular targets but distinct microenvironmental demands. Although preclinical studies dominate orthopedics, their clinical implementation remains limited. However, dental applications have demonstrated superior translational success. Translational convergence between dental osteogenesis and orthopedic regeneration presents a critical pathway for developing clinically viable bone substitutes, requiring interdisciplinary optimization of material-host microenvironment interactions.

From the perspective of development, there is a need to create collagen-based composites with higher strength and improved resistance to degradation for tendon and ligament healing. This can be achieved by incorporating reinforcing phases, such as nano-HAP and silk fibroin, combined with biomimetic fiber arrangement techniques such as counter-flow rotational extrusion and electrospinning. Additionally, optimizing crosslinking techniques, such as the combination of enzymatic and physical crosslinking, can prolong the *in vivo* degradation cycle of collagen, while maintaining its biological activity and providing long-lasting mechanical support. Furthermore, the translation and advancement of more sophisticated collagen scaffold is believed to be optimized in design parameters such as structural strength, biocompatibility and biomechanical performance with the development of artificial intelligence and machine learning. The intelligent system could construct a feedback circle of monitoring structure, extract key features and provide strategies for improvement of resolution and productivity. Therefore, more robust datasets and AI models are needed to predict the formation of bio-scaffold, which would improve the design and application of biomaterials [196].

In conclusion, advancing collagen-based therapies requires balancing material science innovations with the clinical implementation requirements. Interdisciplinary collaboration will drive the development of regulatory-compliant collagen biomaterials that integrate biocompatibility, biomechanical resilience and bioactivity, thereby establishing new paradigms for sports-injury rehabilitation.
